# IVIG-associated anterior uveitis resolving after switch to subcutaneous immunoglobulin

**DOI:** 10.1093/immadv/ltag018

**Published:** 2026-08-11

**Authors:** Vered Schichter Konfino, Oded Shamriz

**Affiliations:** Allergy and Clinical Immunology Unit, Hillel Yaffe Medical Center, Hadera, Israel; Bruce Rappaport Faculty of Medicine, Technion-Israel Institute of Technology, Haifa, Israel; The Lautenberg Center for Immunology and Cancer Research, Institute of Medical Research Israel-Canada (IMRIC), Faculty of Medicine, Hebrew University of Jerusalem, Jerusalem, Israel; Allergy and Clinical Immunology Unit, Department of Medicine, Hadassah Medical Organization, Faculty of Medicine, Hebrew University of Jerusalem, Jerusalem, Israel

**Keywords:** IVIG, uveitis, IVIG-induced uveitis, CVID, SCIG

## Abstract

**Introduction:**

Intravenous immunoglobulin (IVIG) therapy can occasionally induce unexpected inflammatory complications.

**Materials and methods:**

A case report and literature review.

**Results:**

We report a patient with hypogammaglobulinemia and systemic autoimmunity who developed reproducible unilateral anterior uveitis following IVIG administration across multiple formulations. Discontinuation of IVIG resolved the ocular inflammation but caused underlying disease exacerbation. Transition to facilitated subcutaneous immunoglobulin (SCIG) achieved stable serum IgG levels and maintained complete ocular and systemic remission over a four-year follow-up period.

**Conclusion:**

This case demonstrates peak-driven immune activation as a plausible mechanism for IVIG-induced uveitis and highlights SCIG as a safe, effective therapeutic alternative that bypasses rapid peak IgG dynamics while preserving immune protection.

## Graphical Abstract

**Graphical Abstract:**
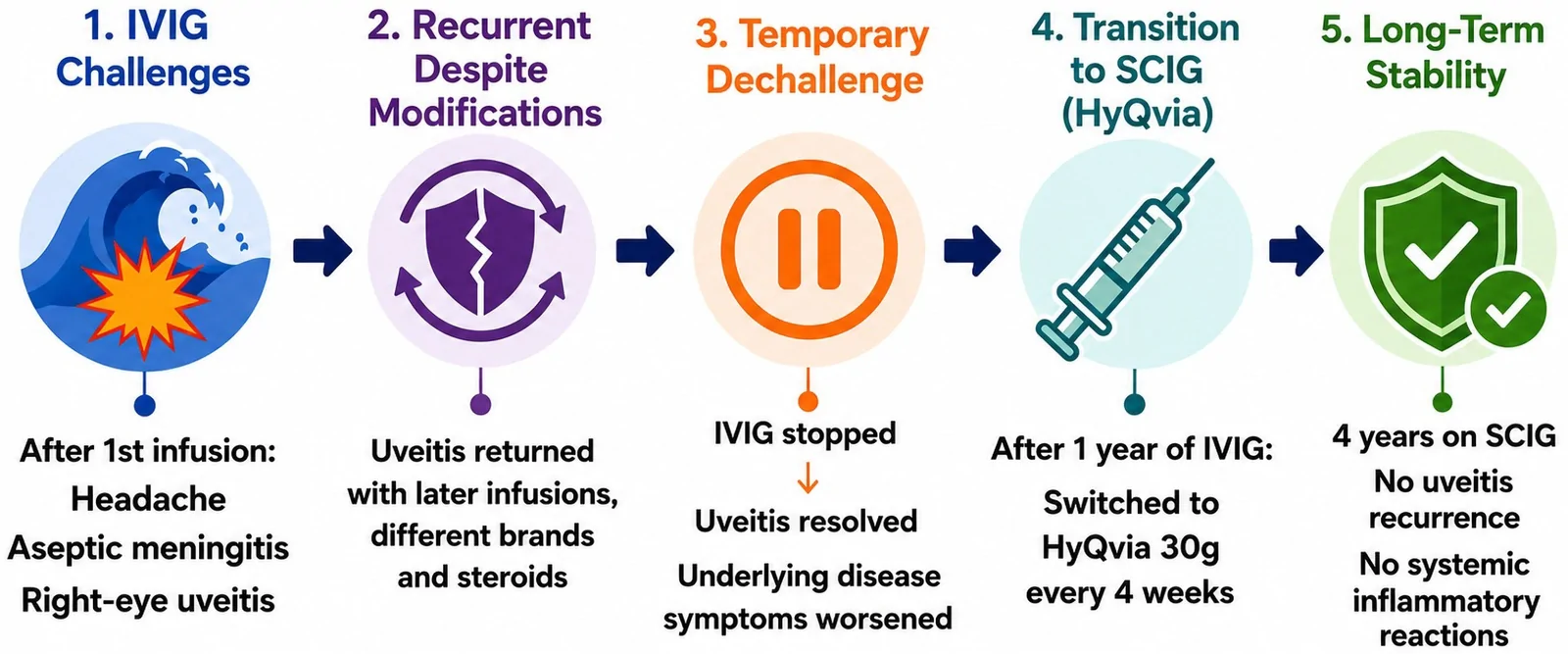

Immunoglobulin (Ig) replacement therapy remains the cornerstone of treatment for common variable immunodeficiency (CVID), with the primary goal of preventing recurrent sinopulmonary infections. Although intravenous immunoglobulins (IVIG) exert predominantly immunomodulatory and anti-inflammatory effects, paradoxical inflammatory adverse reactions have been described, including serum sickness–like reactions, aseptic meningitis, and vasculitis-related phenomena [1, 2]. Ocular inflammatory complications are rare but have been reported. Isolated case reports describe anterior uveitis temporally associated with IVIG administration [3–5]. Drug-induced uveitis is a recognized entity mediated through immune complex formation, cytokine dysregulation, and disruption of immune privilege [6–8]. Herein, we describe a case of reproducible IVIG-associated anterior uveitis with complete resolution following transition to subcutaneous immunoglobulins (SCIG).

A 69-year-old woman was referred for evaluation of persistent hypogammaglobulinemia and multiple autoimmune manifestations. Her medical history was notable for immune thrombocytopenia (ITP; platelet nadir ∼20 000/µL) treated with rituximab, ulcerative colitis (UC) with associated primary sclerosing cholangitis, psoriasis, and papillary thyroid carcinoma status post subtotal thyroidectomy. Ocular history included right-eye vitrectomy for presumed ITP-related hemorrhage and subsequent cataract surgery.

Immunologic evaluation revealed hypogammaglobulinemia, with IgG levels of 336–480 mg/dL (reference: 600–1600 mg/dL), IgA 38 mg/dL (70–400 mg/dL), and IgM 39 mg/dL (30–230 mg/dL). Pre-rituximab IgG levels were unavailable; however, the patient’s baseline calculated globulin level was low at 130 mg/dL, below the literature-reported cutoff of <200 mg/dL suggestive of pre-existing hypogammaglobulinemia [9]. Specific IgG titers to prior vaccinations were impaired for tetanus and *Streptococcus pneumoniae*, although protective titers were observed for measles, mumps, rubella, and varicella. The patient also demonstrated an impaired antibody response to a challenge with pneumococcal polysaccharide vaccine (Pneumovax®), with four-week post-vaccination titers remaining <2 mg/dL. Additionally, CD19^+^ B-cell numbers were preserved, and the CD4:CD8 ratio was elevated at 3.2:1 (normal: 1.8–2.2:1). Rheumatologic testing for autoantibodies was negative. Whole exome sequencing identified no monogenic pathogenic variants associated with hypogammaglobulinemia.

Although the patient’s low baseline globulin, poor vaccine response, severe upper respiratory infections and autoimmune manifestations were suggestive of CVID [10], a definitive diagnosis remains elusive without pre-rituximab immunoglobulin levels to rule out rituximab-induced hypogammaglobulinemia. Regardless, IVIG replacement therapy was started.

Within 48 hours of the first infusion, the patient developed severe headaches, aseptic meningitis, and right-sided anterior uveitis. The ophthalmologic examination of the right eye revealed a pinhole (PH) visual acuity of 6/12, improving to 6/9 with glasses [corrected with glasses (CG)]. Slit-lamp examination demonstrated mild anterior uveitis, characterized by 0.5+ anterior chamber cells and trace (0.5+) flare according to the Standardization of Uveitis Nomenclature (SUN) grading system. Intraocular pressure was within the normal range (15 mmHg). Optical coherence tomography (OCT) of the macula showed no evidence of cystoid macular edema or posterior segment involvement, and fundus examination was unremarkable. Examination of the left eye was unremarkable except for the presence of a cataract with visual acuity of PH 6/7.5 and CG 6/6. The ocular inflammation resolved following treatment with topical corticosteroids and nonsteroidal anti-inflammatory eye drops. A clear temporal association was observed with IVIG rechallenge: recurrent anterior uveitis developed after subsequent infusions, including with different IVIG brands (Gammunex®, intratect® and omnigram®) and premedication with corticosteroids. Temporary discontinuation of IVIG (dechallenge) resulted in complete resolution of the ocular inflammation. However, withdrawal of therapy was associated with worsening of the patient's underlying inflammatory bowel disease, manifested by increased diarrhea, necessitating resumption of immunoglobulin replacement.

One year after initiating IVIG, therapy was transitioned to SCIG (HyQvia®, 30 g every 4 weeks). Notably, IgG trough levels remained stable above 500 mg/dL throughout both IVIG and SCIG therapy. Since then, the patient has been receiving facilitated SCIG for nearly 4 years without adverse events or the need for premedication. Following the transition from IVIG to facilitated SCIG, serial ophthalmologic examinations demonstrated complete resolution of the anterior uveitis. No ocular or systemic inflammatory events recurred during the 4-year follow-up period.

This case highlights a clear, reproducible temporal association between IVIG administration and anterior uveitis, supporting a likely drug-induced inflammatory adverse reaction, with several plausible, although unproven, underlying mechanisms.

A review of the literature (Table 1) [3–5] identified three previously reported patients with IVIG-induced uveitis, including one child and two adults. Two patients received IVIG as replacement therapy for hypogammaglobulinemia, whereas the third received IVIG as immunomodulatory treatment. All three patients were treated with systemic corticosteroids, with clinical improvement in each case. Our patient developed unilateral uveitis despite the systemic administration of IVIG. Interestingly, two of the three previously reported patients also presented with unilateral uveitis following IVIG treatment. One involved a 9-year-old child with X-linked agammaglobulinemia (XLA) who initially developed unilateral uveitis that subsequently progressed to bilateral anterior uveitis. Although speculative, it is possible that our patient’s prior right-eye vitrectomy and cataract surgery predisposed the eye to subsequent inflammation.

**Table 1:** Summary of published reports of IVIG-induced uveitis.

Pt	Age (years)	Sex	Ethnicity	Uveitis type	Indication for IVIG	IVIG brand/dose	Time from IVIG to uveitis	Re-challenge	Treatment (dose)	Outcome	Ref
1	69	F	Jewish	Unilateral	Hypogammaglobulinemia*	Gammunex®/intratect®/omnigram® 30g every 4 weeks	2 days	Yes/recurrence in the same eye	Topical GCS; IVIG switched to SCIG	Complete resolution	Current report
2	44	F	Caucasian	Bilateral acute anterior uveitis	Charcot-Marie-Tooth type 1	Privigen ® 30 g per day for 5 days	2 days	No	Prednisolone (NA)	Visual acuity improvement with traces of anterior uveitis	3
3	9	M	NA	Unilateral followed by a relapse of bilateral chronic anterior uveitis	XLA	Sandoglobulin ® 0.2 g/kg every 2 weeks initially, increased to 0.4 g/kg every 2 weeks	3 months	No recurrence following ANCA-negative Endobulin ®	prednisolone 60 mg cyclosporin (12.5 mg/kg daily for 3 weeks)	Mild anterior uveitis remains	4
4	70	F	NA	Unilateral retinal uveitis	Hypogammaglobulinemia Cipro-resistant *Salmonella enteritidis* in urine and stool	Endobulin ® 10 g/day	7 days	Anterior and posterior uveitis following 1 day of Endobulin ®	GCS (NA)	Clinical improvement	5

CVID, common variable immunodeficiency; F, Female; IVIG, intravenous immunoglobulins; M, Male; NA, data is not available; SCIG, subcutaneous immunoglobulins; XLA, X-linked agammaglobulinemia.

^*^Differential diagnosis between CVID and rituximab-induced hypogammaglobulinemia.

IVIG consists of pooled IgG from thousands of donors and may promote immune complex formation in susceptible individuals. Immune complex deposition and complement activation (C3a, C5a) resemble type III hypersensitivity reactions and may contribute to ocular inflammation [1, 2]. The rapid onset and steroid responsiveness observed in this patient are compatible with such a mechanism.

Although IVIG is generally anti-inflammatory, transient proinflammatory cytokine release has been documented, particularly at higher doses [2, 8]. Acute elevations in IL-6 and TNF-α may promote Th17 differentiation. Th17-mediated immune responses play a central role in uveitis pathogenesis [7], and abrupt cytokine shifts may destabilize ocular immune homeostasis. Additionally, the patient's history of psoriasis suggests that Th17 cell numbers or function may have been elevated prior to IVIG treatment.

The eye represents an immune-privileged site characterized by blood-ocular barrier integrity and local immunoregulatory mechanisms. Disruption of immune privilege is central to uveitis development [7]. Rapid, high serum IgG peaks following IVIG infusion may transiently increase vascular permeability or facilitate immune complex deposition in the uveal microvasculature- mechanisms similar to those proposed for IVIG-associated aseptic meningitis [1, 2]. In this patient, the onset of aseptic meningitis after the first dose, followed by uveitis with subsequent infusions, points to a broader disruption of both central nervous system (CNS) and ocular immune privilege. Given the patient's baseline immune dysregulation, however, IVIG therapy may be a contributing factor rather than the sole primary cause.

Importantly, the absence of recurrence after transition to SCIG provides mechanistic insight. IVIG produces rapid, high-amplitude serum IgG peaks over hours, potentially promoting complement activation and cytokine release. In contrast, SCIG is absorbed gradually via lymphatic pathways, resulting in stable IgG concentrations without abrupt immune activation. This pharmacokinetic distinction supports a model of peak-driven immune dysregulation.

Alternative etiologies, including UC-associated uveitis, were considered. However, the strict temporal reproducibility with infusion and sustained remission under SCIG therapy argue strongly against primary autoimmune ocular disease. Applying the Bradford Hill criteria [11], including temporality, reproducibility, dechallenge response, biological plausibility, and analogy to other IVIG-associated inflammatory events [3–5], supports a potential causal link. However, because these criteria are intended for epidemiological studies rather than single case reports, a definitive causal relationship between IVIG therapy and uveitis remains unproven.

In conclusion, IVIG may rarely induce anterior uveitis through immune-mediated mechanisms in susceptible individuals with immune dysregulation. Rapid high serum IgG peaks may transiently overcome ocular immune privilege, whereas stable pharmacokinetics with SCIG may mitigate this risk. Clinicians should consider IVIG as a potential trigger in new-onset uveitis following infusion and evaluate transition to SCIG when appropriate.

## Data Availability

Data is available upon request from the corresponding author.
